# Supplementary material for: NetR and AttR, Two New Bioinformatic Tools to Integrate Diverse Datasets into Cytoscape Network and Attribute Files
Source: Genes (Basel). 2019 Jun 1;10(6):423. doi: 10.3390/genes10060423 (PMC6628208; doi:10.3390/genes10060423)
Supplement: Supplementary file 1 [file genes-10-00423-s001.zip › Supplementary Materials/Supplementary figure legends.pdf]

Supplementary Figure Legends

Figure S1. Schematic representation of wheel and NetR-integrated networks (supplementary figure). **(a)** Schematic example of a NetR network that combines three individual wheel networks in a manner that intermediate nodes connecting two or more of the wheels have only inbound connections from one or more of the core genes (notice the arrowheads indicating directionality of the connections, cfr. Figure 1b). **(b)** Schematic of a NetR network that incorporates additional genetic and protein interaction data for nodes present in each of the wheel networks, as deposited in InterMine [11,12]. Notice the additional connections from nodes originally on the rim of a wheel network to additional nodes that are brought into the integrated NetR model. **(c)** This procedure expands the set of connections among nodes in the integrated network and, more importantly, can reveal new inbound connections to the original core genes, closing hypothetical loop in the network graph.

Figure S2. The NetR graphical user interface (supplementary figure). **(a-c)** Screen captures depicting a typical command prompt (Windows) or terminal (Mac). To run NetR and AttR, users need to first open a terminal (a), navigate to the local folder where the NetR and AttR scripts have been downloaded (b), and execute the programs (c). From that point onwards, both programs display graphical user interfaces with a familiar and standard look. **(d)** Screenshot of the filtration step in MS Excel to select for Esg DamID targets that were detected in all technical replicates from the original dataset. In this view, the columns for replicates #1 and #2 have already been filtered (notice how there are no data gaps for the first two replicates, and how several row numbers are skipped). The screenshot shows the dialog window that allows users to filter the data

based on the third replicate. **(e)** The filtered data were copied and pasted into a different worksheet, along with the relevant column headings, and subsequently saved as a CSV file.

Figure S3. Processing files to be uploaded to AttR (supplementary figure related to Figure 3). **(a)** Screenshot of the GEO webpage used to obtain the original data on cell type-specific transcriptome profiling (Accession # GSE61361; from the Dutta et al [10]). Scrolling to the bottom of the page shows active links to download the original dataset as a TXT file (Supplementary file *ISC_RNAseq-original.txt*). **(b)** Screenshot of the MS Excel workbook generated by opening the *ISC_RNAseq-original.txt* file directly in MS Excel. The red arrow indicates the placement of a new inserted column that was used to calculate the *ISC average* RPKM values from ISC rep1-3. **(c)** The first four columns from the modified worksheet were copied and pasted as values into a new workbook and saved as a CSV file (Supplementary file *ISC_RNAseq-processed.csv*).

Figure S4. Example of a NetR / AttR network (without InterMine data integration - supplementary figure). **(a)** Screenshot of the file preview window generated by Cytoscape when opening the first network file of a session. By default, Cytoscape had labeled most of the columns as edge attributes and one as interaction type. Clicking on each column header allowed us to re-assign each column as a source node (green circle; *Source Symbol*), source node attribute (green text icon; *Source Primary Identifier* and *Source Secondary Identifier*), interaction type (blue arrow; *Interaction*), edge attribute (blue text icon; n/a), target node (red target icon; *Target Symbol*), target node attribute (red text icon; *Target Secondary Identifier* and *Target Primary Identifier*) or to exclude a column from the mapping (black prohibited icon; n/a). **(b)** Image of the NetR network as displayed immediately after importing it, with node labels added (*View > Show Graphic*

Details). The nodes were displayed as rectangular boxes shaded in light blue, following default display parameters that may be different among users, depending on changes to the default node display parameters inherited from previous Cytoscape sessions. (c) Display of the network after selecting Esg (using the search window on the top/right of the Cytoscape console), and its first neighbors (using the corresponding top icon or *Select* > *Nodes* > *First Neighbors of Selected Nodes* > *Undirected*). The selected nodes will be highlighted in yellow, and their connections to Esg in red, allowing users to move them as a group by a standard click+drag operation. **(d)** View of the network as Esg and all its first neighbors were manually dragged away from the rest of the network. **(e)** View of the network as the selection + dragging operation in (c) and (d) was repeated for Cic and Tis11. **(f)** Screenshot of the Cytoscape file preview window displayed when importing the *ISCattributes-EsgCicTis11net.csv* attributes AttR file. Since AttR generates files in which all columns can be used to map attributes to nodes in the corresponding NetR network and the correct column for mapping keys is pre-selected, all default parameters were accepted by pressing *OK*. (g) Cytoscape dialog window displayed to modify the Node Fill Color parameters when mapping a Continuous attribute. The lowest and highest limits of the continuous range are determined by clicking on the corresponding downward arrows. The adjacent leftward and rightward arrows allow the user to indicate what colors to use for values below the lowest and above the highest limits of the range (in this case, these colors were set to coincide with the limits of the range). An intermediate range value present by default when activating the *Continuous Mapping Editor* was deleted by right-clicking on the corresponding downward arrow. **(h)** Display of the network after the ISC expression data had been imported and mapped to all nodes in the network using *Style* > *Fill Color*:*Mapping Type* > *Continuous Mapping*. Lightest green nodes represent genes that are not expressed in ISCs according to a cell-type specific profiling study by Dutta et al.[10] (average

RPKM value = 0), or genes in the network that were not present in the expression dataset (likely due to annotation updates and mismatches from the publication of the study to date). Darkest green nodes represent those with average RPKM equal to or greater than 50 in ISCs. **(i,j)** Each of the seven clusters in the network were selected manually (by Shift+click-&-drag over a cluster of choice), (i); and their layout was changed using *Layout* > *Grid Layout* > *Selected Nodes Only*, (j).

Figure S5. Example of a NetR / AttR network that integrates InterMine data (supplementary figure). **(a)** Original NetR network obtained by integrating the top 10 targets of Esg, Cic and the interaction data as deposited in InterMine. This network contains several duplicate connections that correspond to physical and or genetic interactions in InterMine, which are redundantly imported into the NetR network. **(b)** Same network as in (a), but with all duplicate interactions removed using a Cytoscape function. **(c-d)** Steps involved in removing terminal nodes and linear paths from the original network. Briefly, a 2-degree filter is imposed on nodes of the network, to select for nodes that have more than one edge (connection) associated to them, as seen in **(f)** (see Methods for details). **(g)** A new network created from the set of filtered nodes selected in (f). A second 2-degree filtration will remove a few more nodes to generate the network shown in **(h)** and **(i)**. In (i), the AttR attributes table was imported into Cytoscape and used to color the nodes in the network according to their gene expression levels in ISCs, as well as display them as ovals when they are classified as ISC homeostasis genes in Gene Ontology (as in Figure 4b).
