# Supplementary material for: NetR and AttR, Two New Bioinformatic Tools to Integrate Diverse Datasets into Cytoscape Network and Attribute Files
Source: Genes (Basel). 2019 Jun 1;10(6):423. doi: 10.3390/genes10060423 (PMC6628208; doi:10.3390/genes10060423)
Supplement: Supplementary file 1 [file genes-10-00423-s001.zip › Supplementary Materials/Tis11-upregulated-original.docx]

|  |  |  |  | RPKPM in sorted cells | | |  | Fold Change | | |
| --- | --- | --- | --- | --- | --- | --- | --- | --- | --- | --- |
| Flybase ID |  | Gene Name |  | Control | Tis11^RNAi^ | Tis11^Flag^ |  | Tis11^Flag^ / WT |  | Tis11^RNAi^ / WT |
|  |  |  |  |  |  |  |  |  |  |  |
| FBgn0050108 |  | CG30108 |  | 22.43 | 11.00 | 33.67 |  | 1.5011992 |  | 0.4904688 |
| FBgn0038490 |  | CG5285 |  | 3.93 | 2.51 | 5.97 |  | 1.51901536 |  | 0.63941925 |
| FBgn0038811 |  | CG4159 |  | 19.89 | 11.81 | 29.86 |  | 1.50156875 |  | 0.59401963 |
| FBgn0031031 |  | CG14218 |  | 1.99 | 0.94 | 6.83 |  | 3.42676106 |  | 0.47100618 |
| FBgn0031032 |  | CG14204 |  | 2.23 | 0.93 | 7.30 |  | 3.27530201 |  | 0.41650753 |
| FBgn0033593 |  | Listericin |  | 19.86 | 1.78 | 36.72 |  | 1.84926154 |  | 0.08972451 |
| FBgn0036057 |  | CG14164 |  | 10.35 | 3.01 | 28.28 |  | 2.73327727 |  | 0.29048061 |
| FBgn0037345 |  | rev7 |  | 28.73 | 11.56 | 61.36 |  | 2.13574807 |  | 0.4024934 |
| FBgn0038302 |  | CG4210 |  | 31.96 | 16.60 | 95.00 |  | 2.97262233 |  | 0.51931833 |
| FBgn0038680 |  | Cyp12a5 |  | 5.81 | 2.39 | 19.34 |  | 3.32776346 |  | 0.41199824 |
| FBgn0040370 |  | CG13375 |  | 6.28 | 4.09 | 15.60 |  | 2.48412735 |  | 0.65134951 |
| FBgn0051259 |  | CG31259 |  | 1.91 | 0.88 | 11.06 |  | 5.79929725 |  | 0.46268775 |
| FBgn0052368 |  | CG32368 |  | 457.67 | 210.81 | 1222.89 |  | 2.67200241 |  | 0.46062648 |
| FBgn0065047 |  | snoRNA:U3:54Ab |  | 5.05 | 0.00 | 12.00 |  | 2.37554098 |  | 0 |
| FBgn0065048 |  | snoRNA:U3:54Aa |  | 5.05 | 0.00 | 12.00 |  | 2.37554098 |  | 0 |
| FBgn0086367 |  | t |  | 17.36 | 6.09 | 37.89 |  | 2.18194802 |  | 0.3508434 |
| FBgn0259710 |  | CG42364 |  | 4.62 | 2.46 | 8.26 |  | 1.79007087 |  | 0.53324768 |
| FBgn0025391 |  | Scgdelta |  | 147.36 | 79.39 | 238.80 |  | 1.62046104 |  | 0.53872546 |
| FBgn0031678 |  | CG31918 |  | 16.55 | 9.27 | 30.62 |  | 1.8495989 |  | 0.55989284 |
| FBgn0035415 |  | CG14966 |  | 16.04 | 8.00 | 35.79 |  | 2.23169629 |  | 0.4990704 |
| FBgn0037709 |  | CG8199 |  | 45.85 | 29.04 | 113.65 |  | 2.47855605 |  | 0.63326434 |
| FBgn0039050 |  | CG17110 |  | 8.15 | 3.12 | 36.40 |  | 4.46482625 |  | 0.38219162 |
| FBgn0039051 |  | CG17109 |  | 109.44 | 30.15 | 266.35 |  | 2.43380727 |  | 0.27552313 |
| FBgn0039052 |  | CG6733 |  | 10.26 | 3.81 | 74.96 |  | 7.30586986 |  | 0.37087098 |
| FBgn0043001 |  | Chrac-16 |  | 13.12 | 7.72 | 33.51 |  | 2.55382071 |  | 0.58822543 |
| FBgn0052695 |  | CG32695 |  | 5.85 | 2.73 | 25.27 |  | 4.31600055 |  | 0.46641023 |
| FBgn0085265 |  | CG34236 |  | 60.35 | 33.84 | 121.22 |  | 2.00863977 |  | 0.56069715 |
| FBgn0262366 |  | CG43064 |  | 22.85 | 8.91 | 87.27 |  | 3.81964408 |  | 0.38994293 |
| FBgn0264437 |  | CR43855 |  | 2.21 | 0.56 | 7.23 |  | 3.27770001 |  | 0.25403953 |
| FBgn0000477 |  | DNaseII |  | 12.62 | 4.55 | 52.22 |  | 4.13682026 |  | 0.36046473 |
| FBgn0001223 |  | Hsp22 |  | 498.61 | 253.92 | 772.86 |  | 1.55001464 |  | 0.50925165 |
| FBgn0001224 |  | Hsp23 |  | 1353.50 | 403.65 | 4418.29 |  | 3.26434429 |  | 0.29822682 |
| FBgn0001995 |  | mRpL4 |  | 31.17 | 19.01 | 66.29 |  | 2.12685253 |  | 0.61003821 |
| FBgn0002031 |  | l(2)37Cc |  | 217.85 | 135.90 | 446.95 |  | 2.0515896 |  | 0.62383064 |
| FBgn0003116 |  | pn |  | 4.72 | 3.11 | 10.58 |  | 2.23922517 |  | 0.65828725 |
| FBgn0003231 |  | ref(2)P |  | 302.41 | 192.01 | 472.57 |  | 1.56265252 |  | 0.63491439 |
| FBgn0003401 |  | shu |  | 12.82 | 7.72 | 31.01 |  | 2.41971956 |  | 0.602067 |
| FBgn0003483 |  | spn-E |  | 4.78 | 2.50 | 18.16 |  | 3.80261434 |  | 0.52368142 |
| FBgn0004187 |  | snRNA:U1:95Cc |  | 3.14 | 1.14 | 7.72 |  | 2.45639441 |  | 0.36270914 |
| FBgn0010278 |  | Ssrp |  | 80.84 | 48.08 | 157.15 |  | 1.94400325 |  | 0.59477316 |
| FBgn0010314 |  | Cks30A |  | 11.05 | 6.21 | 18.22 |  | 1.64855767 |  | 0.56156032 |
| FBgn0010438 |  | mtSSB |  | 87.23 | 57.77 | 156.51 |  | 1.79417985 |  | 0.66229974 |
| FBgn0011787 |  | mRpL12 |  | 92.20 | 45.93 | 155.66 |  | 1.68821379 |  | 0.49813126 |
| FBgn0014033 |  | Sr-CI |  | 6.21 | 3.15 | 17.64 |  | 2.84151342 |  | 0.50676117 |
| FBgn0015299 |  | Ssb-c31a |  | 82.39 | 49.33 | 157.77 |  | 1.915015 |  | 0.59873426 |
| FBgn0015714 |  | Cyp6a17 |  | 51.51 | 20.52 | 101.99 |  | 1.97986186 |  | 0.39839501 |
| FBgn0025592 |  | Gyk |  | 35.36 | 21.16 | 57.45 |  | 1.6244447 |  | 0.5983503 |
| FBgn0025814 |  | Mgstl |  | 108.77 | 65.95 | 224.21 |  | 2.06130367 |  | 0.606317 |
| FBgn0026261 |  | bonsai |  | 52.62 | 29.55 | 85.13 |  | 1.61769557 |  | 0.56156337 |
| FBgn0026737 |  | CG6171 |  | 38.61 | 24.72 | 59.83 |  | 1.54944719 |  | 0.64029483 |
| FBgn0027073 |  | CG4302 |  | 3.09 | 2.01 | 42.73 |  | 13.8459345 |  | 0.65128366 |
| FBgn0027083 |  | Aats-met |  | 8.12 | 4.49 | 18.57 |  | 2.28671864 |  | 0.55341995 |
| FBgn0027359 |  | Tim8 |  | 79.67 | 50.22 | 140.58 |  | 1.764446 |  | 0.63027063 |
| FBgn0028424 |  | JhI-26 |  | 15.97 | 6.92 | 25.00 |  | 1.56567137 |  | 0.43325316 |
| FBgn0029158 |  | Las |  | 20.17 | 13.29 | 40.82 |  | 2.02375711 |  | 0.65910623 |
| FBgn0030026 |  | sni |  | 42.99 | 26.26 | 155.42 |  | 3.61509904 |  | 0.61087023 |
| FBgn0030058 |  | CG11294 |  | 5.39 | 2.04 | 10.56 |  | 1.95836193 |  | 0.37779736 |
| FBgn0030724 |  | Nipsnap |  | 27.67 | 16.89 | 80.47 |  | 2.90807472 |  | 0.61047405 |
| FBgn0031231 |  | mRpL10 |  | 19.79 | 8.21 | 30.73 |  | 1.55293648 |  | 0.41505707 |
| FBgn0032082 |  | CG18088 |  | 2.72 | 0.99 | 13.89 |  | 5.10233011 |  | 0.36256697 |
| FBgn0032160 |  | CG4598 |  | 59.85 | 39.05 | 139.92 |  | 2.33778211 |  | 0.65239192 |
| FBgn0032236 |  | mRpS7 |  | 54.98 | 35.04 | 127.84 |  | 2.32532717 |  | 0.63734915 |
| FBgn0032393 |  | CG12264 |  | 87.09 | 56.61 | 242.20 |  | 2.78108608 |  | 0.65006224 |
| FBgn0032921 |  | Mpp6 |  | 4.95 | 3.17 | 8.17 |  | 1.6491675 |  | 0.64070524 |
| FBgn0033547 |  | CG12935 |  | 17.34 | 10.78 | 32.83 |  | 1.8933301 |  | 0.6218104 |
| FBgn0033883 |  | CG16935 |  | 28.67 | 15.65 | 78.77 |  | 2.74776484 |  | 0.54592928 |
| FBgn0033980 |  | Cyp6a20 |  | 15.46 | 8.51 | 24.08 |  | 1.55834929 |  | 0.55077773 |
| FBgn0034177 |  | CG6796 |  | 5.77 | 3.49 | 22.18 |  | 3.84424362 |  | 0.60395976 |
| FBgn0034605 |  | CG15661 |  | 4.09 | 0.99 | 18.07 |  | 4.42002818 |  | 0.24310889 |
| FBgn0034936 |  | CG2970 |  | 69.46 | 45.29 | 148.92 |  | 2.14406855 |  | 0.65204309 |
| FBgn0034938 |  | CG3803 |  | 16.52 | 9.62 | 35.56 |  | 2.1529743 |  | 0.58261738 |
| FBgn0034950 |  | Pask |  | 5.13 | 3.09 | 8.96 |  | 1.74736912 |  | 0.60192177 |
| FBgn0035309 |  | CG15879 |  | 11.32 | 7.32 | 20.56 |  | 1.81694062 |  | 0.64667197 |
| FBgn0035355 |  | CG16985 |  | 58.97 | 35.52 | 112.76 |  | 1.91213252 |  | 0.60228173 |
| FBgn0035375 |  | pgant6 |  | 43.57 | 19.97 | 162.20 |  | 3.72296986 |  | 0.45826553 |
| FBgn0035529 |  | CG1319 |  | 33.96 | 18.10 | 70.51 |  | 2.07637059 |  | 0.53303336 |
| FBgn0035996 |  | CG3448 |  | 27.54 | 10.84 | 53.66 |  | 1.94837384 |  | 0.39368852 |
| FBgn0036117 |  | CG6321 |  | 11.82 | 7.42 | 23.11 |  | 1.9551519 |  | 0.62808569 |
| FBgn0036126 |  | CG6272 |  | 83.94 | 47.67 | 141.86 |  | 1.68997255 |  | 0.56792937 |
| FBgn0036290 |  | CG10638 |  | 121.82 | 62.71 | 230.39 |  | 1.89131149 |  | 0.51482071 |
| FBgn0036334 |  | CG11267 |  | 369.24 | 224.32 | 917.95 |  | 2.48608068 |  | 0.6075345 |
| FBgn0036702 |  | CG6512 |  | 104.63 | 66.76 | 309.19 |  | 2.95520191 |  | 0.63813524 |
| FBgn0036889 |  | CG14100 |  | 13.23 | 7.63 | 26.55 |  | 2.00684051 |  | 0.57666893 |
| FBgn0036929 |  | CG7668 |  | 34.58 | 18.66 | 80.71 |  | 2.33407849 |  | 0.53959808 |
| FBgn0037018 |  | CG4042 |  | 20.58 | 11.68 | 40.64 |  | 1.97529622 |  | 0.56778837 |
| FBgn0037071 |  | CG7632 |  | 22.74 | 10.77 | 41.15 |  | 1.80920555 |  | 0.47351689 |
| FBgn0037608 |  | mRpL19 |  | 48.11 | 31.69 | 76.77 |  | 1.59594473 |  | 0.6588193 |
| FBgn0037611 |  | CG11755 |  | 10.45 | 6.50 | 24.00 |  | 2.29650078 |  | 0.6216211 |
| FBgn0037637 |  | CG9836 |  | 171.59 | 112.54 | 329.88 |  | 1.9224789 |  | 0.65585808 |
| FBgn0037809 |  | CG12818 |  | 4.84 | 1.97 | 8.28 |  | 1.71021188 |  | 0.40653932 |
| FBgn0037973 |  | CG18547 |  | 98.11 | 34.29 | 208.27 |  | 2.12288965 |  | 0.34951924 |
| FBgn0037974 |  | CG12224 |  | 31.48 | 9.48 | 58.83 |  | 1.86910923 |  | 0.30118189 |
| FBgn0037975 |  | CG3397 |  | 22.59 | 5.74 | 75.28 |  | 3.33312523 |  | 0.25408498 |
| FBgn0038107 |  | CG17327 |  | 94.97 | 51.12 | 159.67 |  | 1.68133504 |  | 0.53830626 |
| FBgn0038292 |  | CG3987 |  | 210.54 | 122.12 | 378.55 |  | 1.79802221 |  | 0.58002356 |
| FBgn0038319 |  | mRpL9 |  | 22.44 | 14.48 | 34.47 |  | 1.53614275 |  | 0.64530634 |
| FBgn0038389 |  | CG5516 |  | 10.05 | 4.73 | 16.67 |  | 1.65848976 |  | 0.47101674 |
| FBgn0038530 |  | AttD |  | 18.24 | 4.45 | 85.92 |  | 4.70963657 |  | 0.24368799 |
| FBgn0038808 |  | Srp14 |  | 54.58 | 27.77 | 86.15 |  | 1.57825052 |  | 0.50875252 |
| FBgn0038974 |  | CG5377 |  | 10.81 | 5.06 | 19.83 |  | 1.83377567 |  | 0.46770948 |
| FBgn0039099 |  | CG10157 |  | 12.75 | 7.46 | 93.62 |  | 7.34368504 |  | 0.58515959 |
| FBgn0039254 |  | Nmnat |  | 16.42 | 8.59 | 26.47 |  | 1.6126012 |  | 0.52328901 |
| FBgn0039766 |  | CG15536 |  | 6.81 | 4.11 | 19.53 |  | 2.86884174 |  | 0.60358098 |
| FBgn0039881 |  | CG1971 |  | 1.55 | 0.87 | 8.09 |  | 5.20868781 |  | 0.56317308 |
| FBgn0040309 |  | Jafrac1 |  | 451.20 | 254.21 | 2108.09 |  | 4.67220599 |  | 0.56341562 |
| FBgn0040928 |  | CG15345 |  | 2.47 | 1.40 | 5.87 |  | 2.38104217 |  | 0.56602236 |
| FBgn0050010 |  | CG30010 |  | 5.56 | 3.62 | 9.40 |  | 1.69033719 |  | 0.65133595 |
| FBgn0050022 |  | CG30022 |  | 72.56 | 37.17 | 126.34 |  | 1.74126604 |  | 0.5122363 |
| FBgn0050100 |  | CG30100 |  | 42.55 | 26.24 | 71.14 |  | 1.6720151 |  | 0.61682135 |
| FBgn0050373 |  | CG30373 |  | 31.19 | 20.67 | 58.87 |  | 1.88728701 |  | 0.66253106 |
| FBgn0050441 |  | CG30441 |  | 1.65 | 0.83 | 7.82 |  | 4.74723773 |  | 0.50414872 |
| FBgn0051133 |  | Slimp |  | 8.90 | 4.51 | 34.51 |  | 3.87718392 |  | 0.5072256 |
| FBgn0051648 |  | CG31648 |  | 13.94 | 8.47 | 22.49 |  | 1.61332989 |  | 0.60736573 |
| FBgn0051740 |  | CG31740 |  | 2.90 | 1.52 | 19.92 |  | 6.8749245 |  | 0.52609435 |
| FBgn0051864 |  | Qtzl |  | 49.08 | 24.49 | 79.08 |  | 1.61133446 |  | 0.49896995 |
| FBgn0051922 |  | CG31922 |  | 8.90 | 5.84 | 20.42 |  | 2.29341823 |  | 0.65650291 |
| FBgn0052500 |  | CG32500 |  | 44.30 | 23.58 | 92.35 |  | 2.0844755 |  | 0.5321862 |
| FBgn0052857 |  | CG32857 |  | 45.06 | 23.16 | 92.06 |  | 2.0429726 |  | 0.51404459 |
| FBgn0053494 |  | CG33494 |  | 4.26 | 1.19 | 17.44 |  | 4.09280799 |  | 0.2782456 |
| FBgn0053502 |  | CG33502 |  | 45.06 | 23.16 | 92.06 |  | 2.0429726 |  | 0.51404459 |
| FBgn0053506 |  | CG33506 |  | 16.60 | 10.73 | 33.67 |  | 2.02865217 |  | 0.64651474 |
| FBgn0053867 |  | His-Psi:CR33867 |  | 7.36 | 2.49 | 14.73 |  | 2.00048618 |  | 0.33871211 |
| FBgn0053926 |  | CG33926 |  | 158.39 | 54.54 | 452.52 |  | 2.85693181 |  | 0.34433024 |
| FBgn0054001 |  | CG34001 |  | 3.65 | 2.42 | 9.37 |  | 2.5702266 |  | 0.66501701 |
| FBgn0067628 |  | CG33331 |  | 5.11 | 2.72 | 23.17 |  | 4.53225463 |  | 0.53121986 |
| FBgn0087021 |  | Spc25 |  | 5.06 | 2.70 | 8.14 |  | 1.61019497 |  | 0.53376037 |
| FBgn0260744 |  | Tango9 |  | 26.06 | 17.04 | 46.17 |  | 1.77178259 |  | 0.65391139 |
| FBgn0260985 |  | RfC4 |  | 38.56 | 21.49 | 69.85 |  | 1.81144049 |  | 0.55737747 |
| FBgn0261373 |  | CG33228 |  | 10.60 | 3.35 | 28.09 |  | 2.65166917 |  | 0.31599796 |
| FBgn0261989 |  | CG42807 |  | 13.03 | 7.83 | 21.69 |  | 1.6652975 |  | 0.60083992 |
| FBgn0262532 |  | CR43086 |  | 2.69 | 0.91 | 14.45 |  | 5.37912495 |  | 0.33777734 |
| FBgn0262563 |  | CG43103 |  | 2.30 | 0.99 | 5.54 |  | 2.41409479 |  | 0.42998898 |
| FBgn0262846 |  | CG43210 |  | 3.37 | 1.32 | 11.89 |  | 3.5315295 |  | 0.39176541 |
| FBgn0262945 |  | CR43264 |  | 64.88 | 40.65 | 102.31 |  | 1.57699469 |  | 0.62660226 |
| FBgn0263981 |  | CR43730 |  | 79.82 | 43.56 | 135.80 |  | 1.70117583 |  | 0.5457092 |
| FBgn0264295 |  | CG43773 |  | 23.12 | 13.77 | 35.81 |  | 1.54869078 |  | 0.59545504 |
| FBgn0264617 |  | CR43957 |  | 5.68 | 2.12 | 14.29 |  | 2.51493286 |  | 0.3733602 |

**Table S2: Positively regulated ISC genes**
