# Supplementary material for: NetR and AttR, Two New Bioinformatic Tools to Integrate Diverse Datasets into Cytoscape Network and Attribute Files
Source: Genes (Basel). 2019 Jun 1;10(6):423. doi: 10.3390/genes10060423 (PMC6628208; doi:10.3390/genes10060423)
Supplement: Supplementary file 1 [file genes-10-00423-s001.zip › Supplementary Materials/Tis11-downregulated-original.docx]

|  |  |  |  | RPKPM in sorted cells | | |  | Fold Change | | |
| --- | --- | --- | --- | --- | --- | --- | --- | --- | --- | --- |
| Flybase ID |  | Gene Name |  | Control | Tis11^RNAi^ | Tis11^Flag^ |  | Tis11^Flag^ / WT |  | Tis11^RNAi^ / WT |
|  |  |  |  |  |  |  |  |  |  |  |
| FBgn0085376 |  | CG34347 |  | 15.57 | 24.41 | 10.10 |  | 0.64838136 |  | 1.56745892 |
| FBgn0085218 |  | CG34189 |  | 4.82 | 11.91 | 1.28 |  | 0.26495576 |  | 2.47378706 |
| FBgn0051076 |  | CG31076 |  | 4.11 | 6.83 | 2.25 |  | 0.54865667 |  | 1.66207246 |
| FBgn0261503 |  | CG42651 |  | 3.61 | 5.59 | 0.93 |  | 0.25807375 |  | 1.54770058 |
| FBgn0002609 |  | HLHm3 |  | 183.53 | 442.28 | 28.49 |  | 0.15525672 |  | 2.40987975 |
| FBgn0033135 |  | Tsp42En |  | 2.02 | 5.02 | 1.05 |  | 0.51768828 |  | 2.48455833 |
| FBgn0036836 |  | CG11619 |  | 10.74 | 18.00 | 4.74 |  | 0.4415721 |  | 1.67595394 |
| FBgn0002732 |  | malpha |  | 194.05 | 479.29 | 97.34 |  | 0.50163049 |  | 2.46992559 |
| FBgn0034154 |  | CG5267 |  | 4.81 | 10.11 | 1.09 |  | 0.22725627 |  | 2.10397193 |
| FBgn0034317 |  | CG14499 |  | 17.54 | 44.94 | 5.28 |  | 0.30107928 |  | 2.56219711 |
| FBgn0042101 |  | CG18744 |  | 3.91 | 7.47 | 2.17 |  | 0.55602282 |  | 1.91022645 |
| FBgn0000451 |  | ect |  | 4.92 | 13.31 | 0.20 |  | 0.04077782 |  | 2.70240469 |
| FBgn0002633 |  | HLHm7 |  | 13.58 | 24.91 | 6.23 |  | 0.4584302 |  | 1.83401674 |
| FBgn0004647 |  | N |  | 24.48 | 41.91 | 15.50 |  | 0.63306155 |  | 1.71202676 |
| FBgn0011695 |  | PebIII |  | 1.90 | 5.67 | 0.47 |  | 0.24703723 |  | 2.99333284 |
| FBgn0030837 |  | CG8661 |  | 6.43 | 19.63 | 3.48 |  | 0.5408433 |  | 3.05220852 |
| FBgn0031045 |  | CG14212 |  | 4.25 | 6.83 | 2.45 |  | 0.57582658 |  | 1.60818105 |
| FBgn0031488 |  | CG17265 |  | 6.22 | 9.89 | 2.83 |  | 0.455746 |  | 1.59077411 |
| FBgn0037753 |  | CG12947 |  | 5.65 | 10.15 | 2.66 |  | 0.47102379 |  | 1.79613084 |
| FBgn0038221 |  | CG3259 |  | 8.15 | 17.32 | 3.89 |  | 0.47726048 |  | 2.12516952 |
| FBgn0040388 |  | boi |  | 5.74 | 11.47 | 3.58 |  | 0.62419037 |  | 1.99769821 |
| FBgn0050280 |  | CG30280 |  | 5.55 | 12.04 | 1.56 |  | 0.28125889 |  | 2.16875173 |
| FBgn0053530 |  | Acp53C14c |  | 2.74 | 5.40 | 1.09 |  | 0.39621898 |  | 1.97138698 |
| FBgn0086693 |  | iav |  | 3.43 | 5.37 | 1.46 |  | 0.42714908 |  | 1.56735037 |
| FBgn0000078 |  | Amy-d |  | 19.84 | 76.93 | 0.39 |  | 0.01940872 |  | 3.87803894 |
| FBgn0000079 |  | Amy-p |  | 47.58 | 153.20 | 0.89 |  | 0.01868439 |  | 3.21959277 |
| FBgn0000097 |  | aop |  | 15.97 | 29.99 | 5.93 |  | 0.3714266 |  | 1.87751396 |
| FBgn0000723 |  | Fps85D |  | 33.61 | 73.30 | 13.55 |  | 0.40314331 |  | 2.18103779 |
| FBgn0002570 |  | Mal-A1 |  | 7.74 | 131.23 | 0.98 |  | 0.12708151 |  | 16.9600809 |
| FBgn0002571 |  | Mal-A3 |  | 4.82 | 11.43 | 3.20 |  | 0.66437927 |  | 2.36964132 |
| FBgn0002735 |  | HLHmgamma |  | 3.47 | 15.15 | 1.77 |  | 0.51054049 |  | 4.36404223 |
| FBgn0003358 |  | Jon99Ci |  | 3.77 | 17.31 | 0.82 |  | 0.21818997 |  | 4.592007 |
| FBgn0003391 |  | shg |  | 47.12 | 77.99 | 22.85 |  | 0.48503834 |  | 1.65522284 |
| FBgn0003716 |  | tkv |  | 53.24 | 93.35 | 19.13 |  | 0.35922286 |  | 1.75334701 |
| FBgn0003863 |  | alphaTry |  | 109.68 | 422.67 | 4.30 |  | 0.03921648 |  | 3.85355202 |
| FBgn0004045 |  | Yp1 |  | 5.94 | 17.30 | 0.73 |  | 0.12220753 |  | 2.91442328 |
| FBgn0004047 |  | Yp3 |  | 10.70 | 23.10 | 1.08 |  | 0.10067172 |  | 2.15808404 |
| FBgn0004108 |  | Nrt |  | 73.48 | 114.25 | 37.04 |  | 0.50408097 |  | 1.55493494 |
| FBgn0004425 |  | LysB |  | 1.74 | 5.25 | 0.95 |  | 0.54181883 |  | 3.00794584 |
| FBgn0004427 |  | LysD |  | 10.50 | 19.11 | 2.73 |  | 0.25989426 |  | 1.82083353 |
| FBgn0004839 |  | otk |  | 3.17 | 10.69 | 1.71 |  | 0.53934988 |  | 3.37268006 |
| FBgn0004860 |  | ph-d |  | 7.23 | 11.45 | 3.68 |  | 0.50908822 |  | 1.58311718 |
| FBgn0004876 |  | cdi |  | 14.18 | 25.91 | 8.17 |  | 0.57626699 |  | 1.82740014 |
| FBgn0005391 |  | Yp2 |  | 9.61 | 24.04 | 0.44 |  | 0.0458305 |  | 2.50086601 |
| FBgn0005592 |  | btl |  | 6.89 | 12.02 | 2.96 |  | 0.42956671 |  | 1.74507754 |
| FBgn0010303 |  | hep |  | 29.45 | 46.08 | 15.87 |  | 0.53869216 |  | 1.56452089 |
| FBgn0010357 |  | betaTry |  | 10.93 | 116.77 | 1.28 |  | 0.11682943 |  | 10.6871619 |
| FBgn0010425 |  | epsilonTry |  | 1.40 | 10.66 | 0.46 |  | 0.32762744 |  | 7.61172705 |
| FBgn0010768 |  | sqz |  | 7.98 | 13.46 | 4.34 |  | 0.54356505 |  | 1.68590266 |
| FBgn0011591 |  | fng |  | 38.70 | 77.03 | 15.63 |  | 0.40387463 |  | 1.99029793 |
| FBgn0012037 |  | Ance |  | 7.49 | 13.10 | 0.94 |  | 0.12566475 |  | 1.74806865 |
| FBgn0013763 |  | CG5210 |  | 1.81 | 6.40 | 0.15 |  | 0.0814778 |  | 3.54038067 |
| FBgn0015039 |  | Cyp9b2 |  | 28.13 | 62.51 | 17.01 |  | 0.6048066 |  | 2.22225185 |
| FBgn0015371 |  | chn |  | 10.21 | 23.15 | 6.76 |  | 0.66235057 |  | 2.26869635 |
| FBgn0016032 |  | lbm |  | 82.66 | 130.32 | 15.15 |  | 0.18324333 |  | 1.57649484 |
| FBgn0016070 |  | smg |  | 10.88 | 17.06 | 5.47 |  | 0.50264917 |  | 1.56843615 |
| FBgn0016123 |  | Aph-4 |  | 2.26 | 8.65 | 0.87 |  | 0.38596907 |  | 3.83184782 |
| FBgn0016797 |  | fz2 |  | 27.71 | 48.84 | 18.03 |  | 0.65079554 |  | 1.76249247 |
| FBgn0020224 |  | Cbl |  | 50.92 | 87.37 | 25.67 |  | 0.50405346 |  | 1.71575863 |
| FBgn0020493 |  | Dad |  | 11.48 | 19.41 | 5.78 |  | 0.50351653 |  | 1.69005216 |
| FBgn0020906 |  | Jon25Bi |  | 2.81 | 22.57 | 0.58 |  | 0.20681565 |  | 8.04330624 |
| FBgn0021776 |  | mira |  | 28.95 | 113.63 | 6.98 |  | 0.24107967 |  | 3.92509741 |
| FBgn0021873 |  | Gef26 |  | 13.33 | 20.35 | 8.66 |  | 0.6494829 |  | 1.52644763 |
| FBgn0023129 |  | aay |  | 25.37 | 53.46 | 12.85 |  | 0.50657291 |  | 2.10709795 |
| FBgn0023197 |  | Jon74E |  | 1.62 | 12.18 | 0.53 |  | 0.32622094 |  | 7.52648299 |
| FBgn0023215 |  | Mnt |  | 23.15 | 36.77 | 10.39 |  | 0.44901336 |  | 1.58830975 |
| FBgn0025454 |  | Cyp6g1 |  | 1.32 | 15.03 | 0.27 |  | 0.20342305 |  | 11.3892321 |
| FBgn0025621 |  | CG16989 |  | 25.62 | 56.03 | 11.79 |  | 0.46003934 |  | 2.18705553 |
| FBgn0026602 |  | Ady43A |  | 11.59 | 30.16 | 7.07 |  | 0.61014026 |  | 2.60302965 |
| FBgn0027499 |  | wde |  | 15.78 | 24.61 | 8.05 |  | 0.510254 |  | 1.55995766 |
| FBgn0027506 |  | EDTP |  | 32.83 | 57.98 | 21.80 |  | 0.66411533 |  | 1.76581974 |
| FBgn0027571 |  | CG3523 |  | 98.04 | 165.22 | 20.95 |  | 0.21368561 |  | 1.68521803 |
| FBgn0029895 |  | CG14441 |  | 20.63 | 43.62 | 11.18 |  | 0.54171554 |  | 2.11438571 |
| FBgn0029994 |  | CG2254 |  | 5.14 | 9.32 | 1.32 |  | 0.25660202 |  | 1.81502279 |
| FBgn0030017 |  | CG2278 |  | 3.27 | 5.17 | 2.10 |  | 0.64281084 |  | 1.57940696 |
| FBgn0030418 |  | CG4004 |  | 5.54 | 10.92 | 3.09 |  | 0.55741792 |  | 1.97154648 |
| FBgn0030452 |  | MFS10 |  | 21.51 | 34.55 | 13.13 |  | 0.61026466 |  | 1.60604427 |
| FBgn0030484 |  | CG1681 |  | 17.33 | 33.99 | 3.68 |  | 0.21222422 |  | 1.96130458 |
| FBgn0030536 |  | CG11068 |  | 4.77 | 9.16 | 2.62 |  | 0.54825002 |  | 1.91907517 |
| FBgn0030688 |  | CG8952 |  | 2.11 | 13.10 | 1.38 |  | 0.65461221 |  | 6.21607904 |
| FBgn0030766 |  | mthl1 |  | 4.32 | 7.20 | 2.87 |  | 0.66588273 |  | 1.66893428 |
| FBgn0030774 |  | spheroide |  | 4.76 | 12.88 | 0.48 |  | 0.0999107 |  | 2.70548089 |
| FBgn0030808 |  | RhoGAP15B |  | 51.02 | 77.85 | 25.78 |  | 0.50521721 |  | 1.52571957 |
| FBgn0030999 |  | Mur18B |  | 1.30 | 18.92 | 0.49 |  | 0.38014735 |  | 14.5976701 |
| FBgn0031078 |  | CG11943 |  | 14.34 | 23.42 | 8.03 |  | 0.55971238 |  | 1.63339006 |
| FBgn0031107 |  | HERC2 |  | 10.79 | 16.88 | 5.38 |  | 0.49907028 |  | 1.56474667 |
| FBgn0031258 |  | CG4297 |  | 10.70 | 22.46 | 6.77 |  | 0.6326051 |  | 2.09974666 |
| FBgn0031654 |  | Jon25Bii |  | 1.73 | 18.58 | 0.00 |  | 0 |  | 10.748173 |
| FBgn0031885 |  | Mnn1 |  | 17.13 | 26.07 | 8.32 |  | 0.48563345 |  | 1.52234718 |
| FBgn0032130 |  | CG3838 |  | 21.70 | 35.57 | 6.03 |  | 0.27783066 |  | 1.63885958 |
| FBgn0032633 |  | Lrch |  | 192.61 | 322.70 | 55.82 |  | 0.28980183 |  | 1.6753594 |
| FBgn0032699 |  | CG10383 |  | 96.36 | 156.12 | 38.88 |  | 0.40347744 |  | 1.62007596 |
| FBgn0032886 |  | CG9328 |  | 10.19 | 22.34 | 4.91 |  | 0.48180024 |  | 2.1929828 |
| FBgn0032899 |  | CG9338 |  | 9.63 | 14.88 | 1.29 |  | 0.13379806 |  | 1.54512315 |
| FBgn0033294 |  | Mal-A4 |  | 9.06 | 15.40 | 1.14 |  | 0.12562084 |  | 1.69969555 |
| FBgn0033297 |  | Mal-A8 |  | 2.77 | 22.39 | 0.13 |  | 0.04544345 |  | 8.08253452 |
| FBgn0033446 |  | CG1648 |  | 90.66 | 156.87 | 37.38 |  | 0.41224385 |  | 1.73022899 |
| FBgn0033638 |  | CG9005 |  | 15.64 | 26.93 | 9.38 |  | 0.59950954 |  | 1.72230073 |
| FBgn0033774 |  | CG12374 |  | 1.84 | 33.66 | 1.10 |  | 0.59532812 |  | 18.2620418 |
| FBgn0033789 |  | CG13324 |  | 5.87 | 8.99 | 3.56 |  | 0.60738941 |  | 1.53264498 |
| FBgn0033820 |  | CG4716 |  | 2.61 | 6.19 | 0.00 |  | 0 |  | 2.37106586 |
| FBgn0034155 |  | unc-104 |  | 56.22 | 142.69 | 11.65 |  | 0.20713337 |  | 2.5380512 |
| FBgn0034247 |  | CG6484 |  | 1.54 | 7.10 | 0.93 |  | 0.60518416 |  | 4.61015529 |
| FBgn0034289 |  | CG10910 |  | 1.42 | 12.45 | 0.06 |  | 0.03906155 |  | 8.79646643 |
| FBgn0034295 |  | CG10911 |  | 54.41 | 186.53 | 5.01 |  | 0.09207727 |  | 3.42797757 |
| FBgn0034570 |  | CG10543 |  | 17.20 | 26.11 | 7.78 |  | 0.45216585 |  | 1.51795525 |
| FBgn0034663 |  | CG4363 |  | 2.86 | 45.56 | 1.61 |  | 0.56344771 |  | 15.9326041 |
| FBgn0034711 |  | CG3290 |  | 3.92 | 8.71 | 0.77 |  | 0.19541872 |  | 2.22167405 |
| FBgn0034817 |  | Art7 |  | 13.66 | 22.16 | 7.95 |  | 0.58210705 |  | 1.6214086 |
| FBgn0034987 |  | CG3363 |  | 6.49 | 11.14 | 3.45 |  | 0.5320913 |  | 1.71680418 |
| FBgn0035360 |  | CG1246 |  | 18.45 | 38.46 | 8.99 |  | 0.4874664 |  | 2.08436308 |
| FBgn0035665 |  | Jon65Aiii |  | 81.22 | 207.77 | 2.15 |  | 0.02645213 |  | 2.55810432 |
| FBgn0035989 |  | CG3967 |  | 11.48 | 19.47 | 5.43 |  | 0.47314394 |  | 1.69612148 |
| FBgn0036004 |  | Jarid2 |  | 15.54 | 25.19 | 10.14 |  | 0.65232943 |  | 1.62044697 |
| FBgn0036106 |  | CG6409 |  | 2.19 | 14.64 | 0.10 |  | 0.04451218 |  | 6.70127766 |
| FBgn0036110 |  | Cpr67Fb |  | 7.65 | 12.22 | 3.11 |  | 0.40716819 |  | 1.59804573 |
| FBgn0036279 |  | Ncc69 |  | 26.10 | 39.77 | 9.59 |  | 0.36735522 |  | 1.52372194 |
| FBgn0036398 |  | CG9007 |  | 15.71 | 25.78 | 9.99 |  | 0.63564251 |  | 1.640571 |
| FBgn0036449 |  | bmm |  | 54.67 | 98.29 | 30.69 |  | 0.56135611 |  | 1.79797971 |
| FBgn0036534 |  | Dcp2 |  | 35.81 | 54.25 | 17.96 |  | 0.50140323 |  | 1.51482115 |
| FBgn0036684 |  | CG3764 |  | 14.87 | 32.42 | 9.14 |  | 0.61460947 |  | 2.18061713 |
| FBgn0036837 |  | CG18135 |  | 70.65 | 108.09 | 31.05 |  | 0.43950455 |  | 1.52987335 |
| FBgn0036948 |  | CG7298 |  | 1.35 | 19.56 | 0.00 |  | 0 |  | 14.5035784 |
| FBgn0037387 |  | CG1213 |  | 7.26 | 12.87 | 3.32 |  | 0.45773813 |  | 1.77355698 |
| FBgn0037633 |  | CG9839 |  | 7.09 | 11.70 | 4.49 |  | 0.63352779 |  | 1.6495904 |
| FBgn0037976 |  | Tk |  | 26.96 | 239.18 | 9.33 |  | 0.34611384 |  | 8.87204407 |
| FBgn0037989 |  | CG14741 |  | 35.25 | 75.13 | 21.34 |  | 0.60529108 |  | 2.13157059 |
| FBgn0038197 |  | foxo |  | 20.46 | 34.20 | 12.47 |  | 0.60971318 |  | 1.67167185 |
| FBgn0038498 |  | beat-IIa |  | 4.67 | 8.88 | 1.16 |  | 0.24769446 |  | 1.90103953 |
| FBgn0038613 |  | Vha100-4 |  | 1.67 | 12.28 | 0.44 |  | 0.26172566 |  | 7.3584917 |
| FBgn0038700 |  | CG3734 |  | 1.56 | 5.76 | 0.69 |  | 0.44429972 |  | 3.68162955 |
| FBgn0039431 |  | CG6490 |  | 5.88 | 11.09 | 3.79 |  | 0.64470774 |  | 1.88695362 |
| FBgn0039471 |  | CG6295 |  | 1.35 | 352.99 | 0.00 |  | 0 |  | 262.243041 |
| FBgn0039474 |  | CG6283 |  | 9.50 | 60.39 | 0.11 |  | 0.01166852 |  | 6.35427419 |
| FBgn0039475 |  | CG6277 |  | 2.94 | 227.44 | 1.33 |  | 0.45236968 |  | 77.4881525 |
| FBgn0039590 |  | CG10011 |  | 9.60 | 19.78 | 6.22 |  | 0.64789727 |  | 2.06098369 |
| FBgn0039747 |  | AdoR |  | 2.35 | 5.50 | 1.04 |  | 0.44327208 |  | 2.34461613 |
| FBgn0039754 |  | CG9747 |  | 2.17 | 6.68 | 0.77 |  | 0.35718234 |  | 3.08032147 |
| FBgn0039808 |  | CG12071 |  | 6.19 | 10.66 | 2.86 |  | 0.4626051 |  | 1.72343679 |
| FBgn0039911 |  | CG1909 |  | 4.13 | 7.40 | 2.63 |  | 0.63586537 |  | 1.78885435 |
| FBgn0040827 |  | CG13315 |  | 7.21 | 14.83 | 1.78 |  | 0.24684779 |  | 2.05644993 |
| FBgn0041182 |  | TepII |  | 49.02 | 101.45 | 28.94 |  | 0.59027238 |  | 2.06942413 |
| FBgn0043471 |  | kappaTry |  | 2.14 | 15.93 | 0.66 |  | 0.30671461 |  | 7.43643356 |
| FBgn0045064 |  | bwa |  | 7.97 | 17.89 | 3.75 |  | 0.47046662 |  | 2.24403793 |
| FBgn0050360 |  | Mal-A6 |  | 14.18 | 49.69 | 0.71 |  | 0.05030758 |  | 3.50371958 |
| FBgn0050460 |  | CG30460 |  | 25.48 | 55.98 | 13.02 |  | 0.51112402 |  | 2.19695537 |
| FBgn0051198 |  | CG31198 |  | 24.62 | 51.17 | 3.63 |  | 0.14759106 |  | 2.0781427 |
| FBgn0051343 |  | CG31343 |  | 3.58 | 17.31 | 2.16 |  | 0.60326784 |  | 4.83712495 |
| FBgn0051632 |  | sens-2 |  | 7.35 | 13.00 | 4.42 |  | 0.60234151 |  | 1.76944335 |
| FBgn0052264 |  | CG32264 |  | 25.77 | 48.47 | 11.71 |  | 0.4542333 |  | 1.88072569 |
| FBgn0052296 |  | Mrtf |  | 9.50 | 14.87 | 5.46 |  | 0.57530761 |  | 1.5657114 |
| FBgn0052407 |  | CG32407 |  | 12.58 | 29.89 | 7.59 |  | 0.60309306 |  | 2.37507749 |
| FBgn0060296 |  | pain |  | 58.22 | 91.77 | 38.07 |  | 0.65385276 |  | 1.57613116 |
| FBgn0082974 |  | snoRNA:Psi28S-3305c |  | 11.84 | 19.57 | 7.70 |  | 0.64990921 |  | 1.65281097 |
| FBgn0085285 |  | CG34256 |  | 8.13 | 17.36 | 3.23 |  | 0.39779067 |  | 2.13579526 |
| FBgn0085430 |  | CG34401 |  | 24.27 | 38.00 | 15.28 |  | 0.62959118 |  | 1.56540637 |
| FBgn0259176 |  | bun |  | 377.19 | 567.77 | 163.41 |  | 0.43324514 |  | 1.50528387 |
| FBgn0259738 |  | CG42392 |  | 3.05 | 10.79 | 0.00 |  | 0 |  | 3.53787951 |
| FBgn0259967 |  | Sfp53D |  | 2.62 | 5.51 | 1.04 |  | 0.395917 |  | 2.09858108 |
| FBgn0261444 |  | CG3638 |  | 87.76 | 134.53 | 53.81 |  | 0.61309325 |  | 1.53279864 |
| FBgn0261703 |  | gce |  | 19.37 | 36.16 | 12.77 |  | 0.6592736 |  | 1.86671313 |
| FBgn0262127 |  | kibra |  | 36.68 | 58.48 | 17.49 |  | 0.47689388 |  | 1.59449379 |
| FBgn0262160 |  | CG9932 |  | 3.30 | 5.19 | 1.97 |  | 0.59521034 |  | 1.57014184 |
| FBgn0262624 |  | Tmhs |  | 9.32 | 25.45 | 4.16 |  | 0.44691319 |  | 2.73154941 |
| FBgn0263593 |  | Lpin |  | 90.91 | 150.19 | 50.84 |  | 0.55923763 |  | 1.65211499 |
| FBgn0263773 |  | fok |  | 105.29 | 187.52 | 60.04 |  | 0.57028721 |  | 1.78102918 |
| FBgn0263873 |  | sick |  | 7.73 | 16.20 | 4.34 |  | 0.5615733 |  | 2.09504388 |
| FBgn0264273 |  | Sema-2b |  | 7.58 | 11.98 | 1.64 |  | 0.21665117 |  | 1.57988302 |
| FBgn0025712 |  | CG13920 |  | 3.91 | 5.89 | 1.91 |  | 0.48958184 |  | 1.50875689 |
| FBgn0032901 |  | sky |  | 36.99 | 55.61 | 20.69 |  | 0.55928419 |  | 1.50332495 |
| FBgn0259745 |  | wech |  | 12.90 | 19.40 | 6.66 |  | 0.51615166 |  | 1.50353802 |

**Table S1: Negatively Regulated ISC Genes**
