# Supplementary material for: NetR and AttR, Two New Bioinformatic Tools to Integrate Diverse Datasets into Cytoscape Network and Attribute Files
Source: Genes (Basel). 2019 Jun 1;10(6):423. doi: 10.3390/genes10060423 (PMC6628208; doi:10.3390/genes10060423)
Supplement: Supplementary file 1 [file genes-10-00423-s001.zip › Supplementary Materials/Supplementary Methods.pdf]

Supplementary Methods

The following contains a full version of the methods used in this manuscript and represents an expanded version of the Methods section in the main text of the manuscript.

Installing NetR, AttR and their dependencies. NetR and AttR were coded in Python3 and need to be started from a command terminal. Python 3 can be downloaded and installed from the Python website (https://www.python.org/).

Before using NetR and AttR for the first time, users also need to install two modules (dependencies) that are used by both programs: the Intermine Application Programming Interface (API) (https://www.programmableweb.com/api/intermine), and the Pandas package for data structure and analysis for Python (https://pandas.pydata.org). Both dependencies need to be installed through a command terminal. To access a command terminal, Mac OS users can search for "terminal" and Windows users can search for "command prompt" using their operating system search tools. The "Terminal" (Mac) and "Command prompt" (Windows) applications will likely be presented as top choices. Clicking on them will open a command terminal (Figure S2a) mapped to a home folder. To install the Intermine API and Pandas, Windows users should type "pip install [intermine | pandas]" and Mac users should type "pip3 install [intermine | pandas]" in the terminal, respectively, and Enter/Return (a message may appear warning users about an outdated version of pip3 – but they can be ignored, as this should not affect the installation of both dependencies).

The NetR and AttR scripts (*NetR.py* and *AttR.py*) are available as Supplementary Files and have been deposited in a GitHub repository (https://github.com/armenhalajyan/NetRAttR). We recommend that users download and save both files locally in an easily accessible folder (e.g. a folder called "NetrR_AttR" in their Desktops). Once Python3, the Intermine API and Pandas are

installed, NetR and AttR can be started from the terminal. Users first need to navigate to where the NetR and AttR scripts were saved. If saved to a folder named "NetR_AttR" in the desktop, users first navigate to the desktop, by entering "cd Desktop" in the terminal, followed by "cd NetR_AttR" (Figure S2b). Once in the correct folder, either program can be started by entering "python3 [NetR.py | AttR.py]" (Mac users) or "py [NetR.py | AttR.py]" (Windows users) which will open the corresponding program graphic user interfaces (GUI, Figure S2c), which function with a standard look and operability. Alternatively, Windows users can download executable files for both programs from the same GitHub repository, and start the programs by double-clicking on the file or the program icons (which will be displayed when the executable files have been copied to a Desktop).

Processing files for use with NetR. The sample files used in this manuscript are available as supplementary material, both in their original versions as obtained from their corresponding source, as well as processed CSV files ready to be used with NetR and AttR.

The list of putative targets of Esg in intestinal cells as identified by DamID was originally published as an MS Excel file in the original article [7] (Supplementary Table S2; *Esg_DamID data-original.xlsx*). The original file lists 2327 putative targets of Esg; however, these raw data correspond to all genes that were identified as putative targets in one or more of the DamID technical replicates. In order to select only for genes that were targeted by Esg in all technical replicates, the Max Avg ratio columns (F, H, J) were filtered for cells that contained a ratio equal to or greater than 2. To perform this filtering operation, all columns (F thru K) were highlighted and the Filter button under the Data tab clicked, which generates small downward arrowheads next to each column heading. Clicking on the arrows associated with each Max Avg ratio column

displayed a filtering menu, where the "Greater than or equal to = 2" filter was set (Figure S2d). Next, all filtered cells we selected, copied and pasted into a new MS Excel workbook. To restore the column headings (that had been hidden by the previous filtration), all filters were cleared and the cells containing the relevant column headings were manually selected, copied and pasted in the top row in the new workbook (Figure S2e). The new workbook was then saved as a Comma-separated values (csv) file and contained only 1071 genes from the original target list (*Esg_DamID data-processed.csv*). To obtain a list of the top 10 Esg targets used to illustrate the integration of InterMine data, *Esg_DamID data-processed.csv* was opened in MS Excel and a new column was inserted between columns E (Chromosome) and F (Max avg ratio) and named "Mean ratio". This new column was then used to calculate an average of the intensity ratios from each technical triplicate in all rows. The entire dataset was sorted in descending order by the Mean ratios (Data>Sort>Sort by:Mean ratio>Values>Largest to smallest; My list has headers:check). The column headers and the first ten data rows were copied into a new MS Excel workbook and saved as a CSV file (*Esg_DamID data-top10.csv*).

The list of genes differentially expressed in intestinal stem cells following the overexpression or knockdown of the RNA-binding protein Tis11 was obtained from two supplementary MS Word files associated with the original article [9] (Supplementary File 3 and 4; Tis11-downregulated-original.docx and Tis11-upregulated-original.docx). Each of these MS Word documents contained a table and a bottom caption. To generate the CSV file to be used with NetR, the cells corresponding to the FlyBase ID's and Gene names for the downregulated genes were copied into an empty excel sheet under the corresponding headers. This operation was repeated for the MS Word file for genes positively regulated by Tis11, and the values were pasted immediately following the end of the list of downregulated genes. An unnecessary blank column that was

present in both MS Word tables was removed, and the MS Excel workbook was saved again as a CSV file (Tis11-diff expressed -processed.csv).

Lastly, we used supplementary data from a report by Jin and collaborators, who used DamID in whole midguts to generate a list of putative Capicua binding sites [8] (S1 Table; Cic_DamID data-original.xlsx). The original MS Excel file contained 2 worksheets, but only data from the "CicDam" worksheet were used, from which the Gene ID and Symbol columns (including headers) were copied and pasted into a new MS Excel workbook and saved as a CSV file (Cic_DamID data-processed.csv). To generate the list of top 10 Cic targets used to illustrate the integration of InterMine data, the original Cic_DamID data-original.xlsx was opened in MS Excel. On the CicDam worksheet, all data rows were sorted in descending order based on their "Log2 fold change of CicDam/Dam-only" values (Data>Sort>Sort by:column E>Values>Largest to smallest; My list has headers:uncheck). The top 10 rows were selected, copied to a new workbook and saved as a CSV file (*Cic_DamID data-top10.csv*).

Uploading datasets to NetR. When users start NetR, they will see a graphical user interface (GUI) like the one shown in Figure 2a. On the first row, the user first chooses an arbitrary "Dataset name" to describe concisely the nature of the uploaded data. Users may choose any name for each uploaded dataset, since these names are only used to keep track of uploaded datasets; however, it is recommended that the *Dataset name* include a reference to the core gene/protein, the technique used to generate the data and any other relevant information specific to the way that the dataset was obtained. For example, we chose "Esg-DamID" as the *Dataset name* for the list of putative targets of Esg in the Supplementary file *Esg_DamID data-processed.csv*.

On the second row, the "Open File" button triggers a standard file manager dialog box that allows the user to browse through their local files to select the one containing a list of genes or proteins to use with NetR. Once the file is selected, the text field next to the *Open File* button will display the path to that file.

On the third row, there is a drop-down menu with options for organism names. Each name is linked to an InterMine database with information about that particular species. The species displayed by default is *Drosophila melanogaster*, but NetR can also work with data from the nematode *Caenorhabditis elegans*, *Danio rerio* (zebrafish), *Homo sapiens* (humans), *Mus musculus* (mouse) and *Rattus norvegicus* (rat).

On the fourth and fifth rows, other text entry fields prompt the user to type the name of the core gene or protein, and the technique used to generate the uploaded data, respectively. The latter is used by NetR to populate the "Interaction" column in the output file, which Cytoscape will later use to display different connection (edge) types. For our example, the *Core gene symbol* would be "esg", and the *Technique*, "DamID". It is critical to type in the correctly and completely spelled gene symbol in this window. NetR will use the typed symbol to communicate with the InterMine database, and if the symbol is not properly spelled the program cannot proceed, forcing a re-initiation of the process. In such a case the user will be notified and asked to provide a valid core gene identifier. When the user clicks "Okay" the text entry fields are cleared, and the user can fill them in with the correct information.

A checkbox labeled "*Check if the file has a header*" allows the user to determine if the values in the first row of a list or table should be used as column headers. If this box is left unchecked, all rows in a list or table will be treated as data and the columns will be numbered instead of being named. The last row contains the remaining three buttons: "*Reset*", "*Clear*" and "*Submit*". The

Reset button restarts the program and discards any submitted datasets to that point, whereas the *Clear* button clears only the text entries for *Dataset name*, *Core gene symbol* and *Technique windows*, allowing the user to reenter the information about the current dataset (if users chose to discard the current dataset, they would do so by re-routing NetR through a new file path using the *Open file* button).

Clicking on the *Submit* button opens a read-only preview of the selected CSV file, displaying column headers and the first five rows of the table (Figure 2b). The column headers are active buttons that allow users to select or unselect columns that contain genes or proteins connected to the core gene or protein (when a column is selected, the column label becomes bold and underlined; clicking again will unselect the column). Users will usually need to select only one column containing the relevant data; however, NetR also allows users to select more than one column if the original file contained complementary lists of targets corresponding to the same core gene or protein. When the desired column(s) are selected, the user clicks "*Okay*" and NetR uploads the chosen data into the program. As part of the first data upload, a dialog box will ask users if they want to integrate interaction data from InterMine (Figure 2c). Users will have to answer the question only once, and their choice will apply to all the datasets uploaded in the current NetR session.

Processing files for use with AttR. As a first set of attributes for our NetR networks, we used data from Dutta *et al.* [10], who profiled gene expression by RNA-sequencing from different cell types and from different regions of the *Drosophila* intestinal epithelium, under homeostasis or following infection (GEO Accession # GSE61361, Figure S3a; Supplementary file *ISC_RNAseq-original.txt*). The original dataset was downloaded in TXT format and opened directly in MS

Excel, showing 3 columns with gene identifier information, followed by several columns grouped into three sections: *Physiological*, *Infection* and *Regions* (Figure S3b). The top row was deleted and a new column (ISC average) was inserted before the three columns for ISC RPKM, which was used to calculate the mean of the corresponding RPKM values using a standard MS Excel AVERAGE formula. The first four columns (GeneID, ANNOTATION_SYMBOL, NAME, and the new column ISC average) were then copied, pasted as values into a new workbook (Figure S3c) and saved as a CSV file (Supplementary file *ISC_RNAseq-processed.csv*).

In addition to the Discrete/Continuous attributes table from RNA-seq data described above, a List attributes file was generated based on a classification of *Drosophila* genes under the Biological Process "Intestinal stem cell homeostasis" in the Gene Ontology Consortium (http://www.geneontology.org/) [19,20]. The original list of genes was obtained by typing "intestinal stem cell homeostasis" in the Search GO data window and then clicking on the Genes and gene products button. The search was then narrowed down to retrieve only fruit fly genes directly annotated under "Intestinal stem cell homeostasis", by applying the *Organism:Drosophila melanogaster* and Direct annotation:Instestinal stem cell homeostasis filters on the left side panel. The filtered list contains 30 genes and was downloaded using the Custom DL button (using "Gene/product (bioentity_label)", "Gene/product name (bioentity_name)" and "Organism (taxon_label)" as selected fields to download). Clicking on *Download* generated a plain text webpage with the 30 genes in the list, which was saved as a TXT file from browser, imported into a new MS Excel workbook and saved as a CSV file (Supplementary files; *GO_IntestinalHomeostasis.csv*).

Uploading datasets to AttR. To run AttR, users can download the AttR Python script provided as supplementary file (*AttR.py*) and start the program from a command prompt terminal as described above. The main window of AttR in shown in Figure 3a. On the first row, there is an "*Organism*" drop-down menu that serves the same purpose as the equivalent menu on the NetR interface. On the second and third rows, two "*Open file*" buttons allow users to find and select the NetR CSV file to which they want to assign an attributes dataset and the corresponding dataset file, respectively. Once the NetR and attributes file are selected, the text entry fields will display the path to the chosen files. Note that users can upload an attributes file that has been previously generated with AttR, which allows users to add new attributes to an expanding collection of attributes for their network (for instance, if new attributes data of interest become available through publication).

Next, users determine whether the attributes dataset to be uploaded is in List or Discrete/Continuous Values format, respectively. Finally, there is a checkbox to indicate if the attributes dataset to be uploaded has headers. If users leave this box unchecked, all rows will be treated as valid data, and the user will need to provide names for the columns which they wish to include in the output.

The bottom row contains 3 buttons: "*Reset*", "*Clear*" and "*Submit*". The *Reset* button restarts the program, and any submitted datasets up to that point are lost. This is useful if the user has made a mistake in submitting any of the previous datasets. The *Clear* button clears all selections and allowing the user to reenter the information about the current dataset.

When users click *Submit*, AttR opens a preview of the selected attribute CSV file, displaying the first few rows of the uploaded table as read-only. Depending on the type of attributes dataset selected by the user, the file preview window is slightly different. If the user has selected

Discrete/*Continuous* attributes, the header row in the preview window will have choice buttons associated to text entry fields in each column (Figure 3b). Using the choice buttons, the user indicates which of the columns should be treated as the "Mapping Key", i.e. the column which contains the gene identifiers to which the values in the remaining columns correspond. If the user has indicated that the file has a header row, the text entry fields will be automatically populated with the values in the first row of the file; otherwise they will be left empty. The user may edit the column names using the text entry fields or clear the text entry field if they want to exclude a given column from the output attribute table (e.g. in Figure 3b, the header "Name" was deleted from the text field, which excludes the corresponding column from further AttR processing). The column names in the text entry fields will be used as attribute names.

If the user has selected *List* attributes, the preview window will have a header row, consisting of text entry fields each column in the attribute CSV file and the first 5 rows of data and an "Okay" button (e.g. Figure 3c). Note that the column headers will be empty or will have been automatically populated, depending on whether users ticked the "Check if the attribute has a header" box. If empty, the user needs to type in the attribute name on the corresponding column header that lists all the genes with such attribute (e.g. we typed "ISC Homeostasis" in the column that lists all the genes that were classified under this category by Gene Ontology). Conversely, if users would like to ignore a column proceeding further, they should simply clear the corresponding header text entry box, since all columns with empty boxes will be ignored in subsequent steps.

When the user presses "Okay" in the file preview window, the program begins processing the submitted data. This may take anywhere from a few seconds to a few minutes. When done, the program will display a message box asking the user whether they would like to add another attribute table. If the user answers "Yes", the dropdown menu and the Open File button on the first

and second rows are disabled (which fixes the Organism and NetR file choices), while the remaining selections are cleared to allow the user to make the appropriate selections for the next data set. When a user answers "No" to uploading any additional attribute files, the program begins combining the different attribute datasets. This may take from a few seconds to a few minutes. AttR uses the submitted datasets to update the gene/protein identifiers through the selected InterMine database and outputs the combined attribute table as Discrete/Continuous attribute dataset in CSV format. A file dialog window will ask the user to name and choose a location to save the output CSV file.

Mapping NetR networks in Cytoscape. The *EsgCicTis11_noIM.csv* file was opened in Cytoscape v3.7.1 (File > Import > Network from File > (…*EsgCicTis11_noIM.csv*…) > Open). Once the NetR file is selected, Cytoscape displays a preview of the table that is used to determine which columns will be used as source or target nodes, interactions (edges), as well as their attributes. The "Source Symbol" was designed as the "Source Node" column (green dot icon); "Interaction Type" was selected as the "Interaction" column (blue arrowhead icon); and "Target Symbol" was selected as the "Target Node" column (red target icon). The "Source Primary Identifier" and "Source Secondary Identifier" were assigned the "Source Node Attribute" column types (green text icon), whereas "Target Secondary Identifier" and "Target Primary Identifier" were assigned as "Target Node Attribute" columns (red text icon), respectively (Figure S4a). Clicking "OK" opened the network. By default, the network did not show node labels; they were displayed via View > Show Graphic Details (Figure S4b).

The Search function in Cytoscape was used to select each of the core genes sequentially (Esg, Cic, Tis11). After each core gene was selected (and highlighted in yellow on the network view),

their first neighbors were also added to the selection by clicking on the First Neighbors of Selected Nodes button on the top banner (adjacent houses icon) or Select > Nodes > First Neighbors of Selected Nodes > Undirected. Once the combination of core gene+first neighbors was selected (e.g. for Esg, Figure S4c), they were all manually dragged to a side of the map (Figure S4d). Repeating the procedure for each of the core genes resulted in seven distinct clusters: 3 clusters of genes targeted by only one of the core genes, 3 clusters of genes targeted by two core genes and a single cluster of genes targeted by all three core genes (Figure S4e).

Next, the *ISCattributes-EsgCicTis11net.csv* file generated by AttR was imported as a node attributes file, by clicking the "Import table from file" button from the top panel (or File > Import > Table from file…) and selecting the appropriate AttR file. Cytoscape displayed a preview window where the "Mapping Key" and the columns to upload need to be selected (Figure S4f). Usually, the correct mapping key is already selected when working with AttR files, and all the additional columns can be selected to be imported as attributes; therefore, users can directly click *OK* (or uncheck some of the unnecessary columns before clicking *OK*). The previous step loads the attributes file into memory; to apply those attributes to nodes and interactions in a network the Node and Edge Styles need to be changed. In the *Style* tab, *Fill Color:Mapping Type* was set to Continuous Mapping, and the newly imported "ISC" column was selected for mapping (Column:ISC). Double-clicking on the gradient shown in the "Fill Color" drop-down box activates the *Continuous Mapping Editor for Node Fill Color*. The limits of the range were changed under *Set Min and Max…* to 0-50. Then, the color for the lower and upper limits of the range were changed by double-clicking on the leftmost and rightmost downwards arrows of the range (Figure S4g). Under *Colors>RGB*, the *Color Code* were set to "99FF99" (light green) and "006600" (dark green), respectively. The middle downward arrow was deleted from the range, and the leftward

and rightward arrows were set to match the limits of the range, respectively. Not all genes in the network were present in the dataset from Dutta et al. [10] used to generate the ISC attributes file. These genes were forced to display the light green color for no/low expression by clicking on the Default ("*Def*") box under the *Fill Color* submenu (RGB > Color Code: 99FF99). Applying the Node Style settings described above led to a network in which nodes displayed a color that correlates with their level of expression in ISCs (Figure S4h). To allow for a better contrast of the node labels, the settings for *Label Color* were also modified as follows: *Mapping Type* and *Column* were set to "Continuous Mapping" and "ISC" respectively, as before. In the *Continuous Mapping Editor for Node Label Color*, the *Set Min and Max* range was again set to 0-50. Double-clicking on the leftward arrowhead for "below color" and on the downward arrowheads at the 0 and 25 positions, the label color was set to black (*Colors>RGB>Color Code* "000000"). Similarly, the downward arrowhead at position 50 and the rightward arrowhead for "above color" were set to Color Code "FFFF00" (yellow). Lastly, the parameters for *Shape* were modified in the Control Panel to incorporate the GO classification under Intestinal stem cell homeostasis: Control Panel > Style (Nodes) > Shape > Mapping Type : Discrete; Column : ISC Homeostasis; false : Round Rectangle; true : Ellipse.

To obtain the network image shown in Figure 4a, two final display modifications were applied. First, each cluster was selected manually (Shift + mouse click and drag over desired cluster – Figure S4i) and a Grid layout was applied (*Layout > Grid Layout > Selected Nodes Only* – Figure S4j). This procedure was repeated for all clusters, followed by additional slight re-arrangements of the distribution of clusters in the network. Second, the Esg, Cic and Tis11 nodes were enlarged for illustration purposes. Each node was selected using the Search window, and their default display parameters for *Height*, *Width* and *Label Font Size* were bypassed under the Style tab

(clicking on the "Byp" box of each category, *Height* was set to 140, *Width* to 450 and *Label Font Size* to 240).

Filtering out terminal nodes and linear paths. The *Esg_Cic-top10_IM.csv* NetR file was imported to Cytoscape (Figure S5a). First, its duplicated edges (connections) were removed. Clicking on *Edit* > *Remove Duplicated Edges…* opens a dialog window where the corresponding network was selected. Both *Ignore Edge Direction* and *Create an edge table column with number of duplicated edges* were checked (Figure S5b). Then a 2-step node filtration was performed to discard any terminal or intermediate nodes. Nodes with degrees equal to or greater than 2 were selected through the *Select* tab under the *Control panel*. A new filter was created and named "2-edges or more" (*Options* button > Create new filter – Figure S5c). Using the *Add new condition* button (+) and selecting "Degree Filter" (Figure S5d), a filter was set with the options In+out : Is : between : 2 : and : 200 (an arbitrary large number) : inclusive (Figure S5e). All nodes with 2 connections or more became highlighted in yellow (Figure S5f). A new network was then created from the selected nodes (*File* > *New Network* > *From Selected Nodes, All Edges*), which effectively filtered out terminal nodes with only one connection (Figure S5g). The same filtration step was repeated on this intermediate network (*Select* > *2-edges or more* > *Apply*), which effectively filtered out nodes that originally linked a core gene and terminal nodes and which had become terminal nodes in the intermediate network. A new network was once again created based on the selected nodes following the second filtration step (Figure S5h).

The *ISCattributes-EsgCicIMnet.csv* AttR file was then imported onto this new network as before, and the node display modifications described above were used to color nodes based on their ISC expression, as well as modify their shape based on GO classification (Figure S5i). To

obtain the network in Figure 5b, nodes were manually rearranged, and the Edge Style parameters were modified as follows: Control Panel > Style > Edge > Stroke color > Mapping Type : Discrete Mapping; Column : Interaction; DamID : RGB 0000FF; genetic : RGB 00FF00; physical : RGB FF00FF.

Statistical analysis. Hypergeometric tests were conducted using the dhyper function in an *ad hoc* R script (supplementary file *50+esg-full-cic-tis11 no intermine.R*), in which the number of rows in the RNA-seq table was used as the number of all drosophila genes (15682), while the number of genes for each wheel (Esg: 1072; Cic: 3693; Tis11: 309), the entire network (4459) and the shared targets (23) were acquired by selecting the respective group of nodes in Cytoscape and recording the number of selected nodes. The number of "highly expressed" genes in intestinal stem cells was calculated as the number of rows in the RNA-seq table on which the ISC average RPKM was above 50. The number of "highly expressed" genes in a network and its various subsets was calculated by using a column filter in Cytoscape (Contro Panel > Add new condition… > Column filter > Choose column…: ISC > between 50 and 50,000 - arbitrary large number). The number of selected nodes was recorded in the R script. The number of "not highly expressed" genes was calculated in each case by subtracting the number of "highly expressed" genes from the total number of genes in a set. Finally, to calculate the p-values for each reported group, the "dhyper" function was used in R. For instance, when testing the Esg wheel vs. the Drosophila genome, the number highly expressed and not highly expressed genes in the Esg wheel were 155 and 896 respectively, whereas the highly expressed and not highly expressed genes across the *Drosophila* genome were 1251 and 14431 respectively.
