# Supplementary figures and images for: NetR and AttR, Two New Bioinformatic Tools to Integrate Diverse Datasets into Cytoscape Network and Attribute Files
Source: Genes (Basel). 2019 Jun 1;10(6):423. doi: 10.3390/genes10060423 (PMC6628208; doi:10.3390/genes10060423)

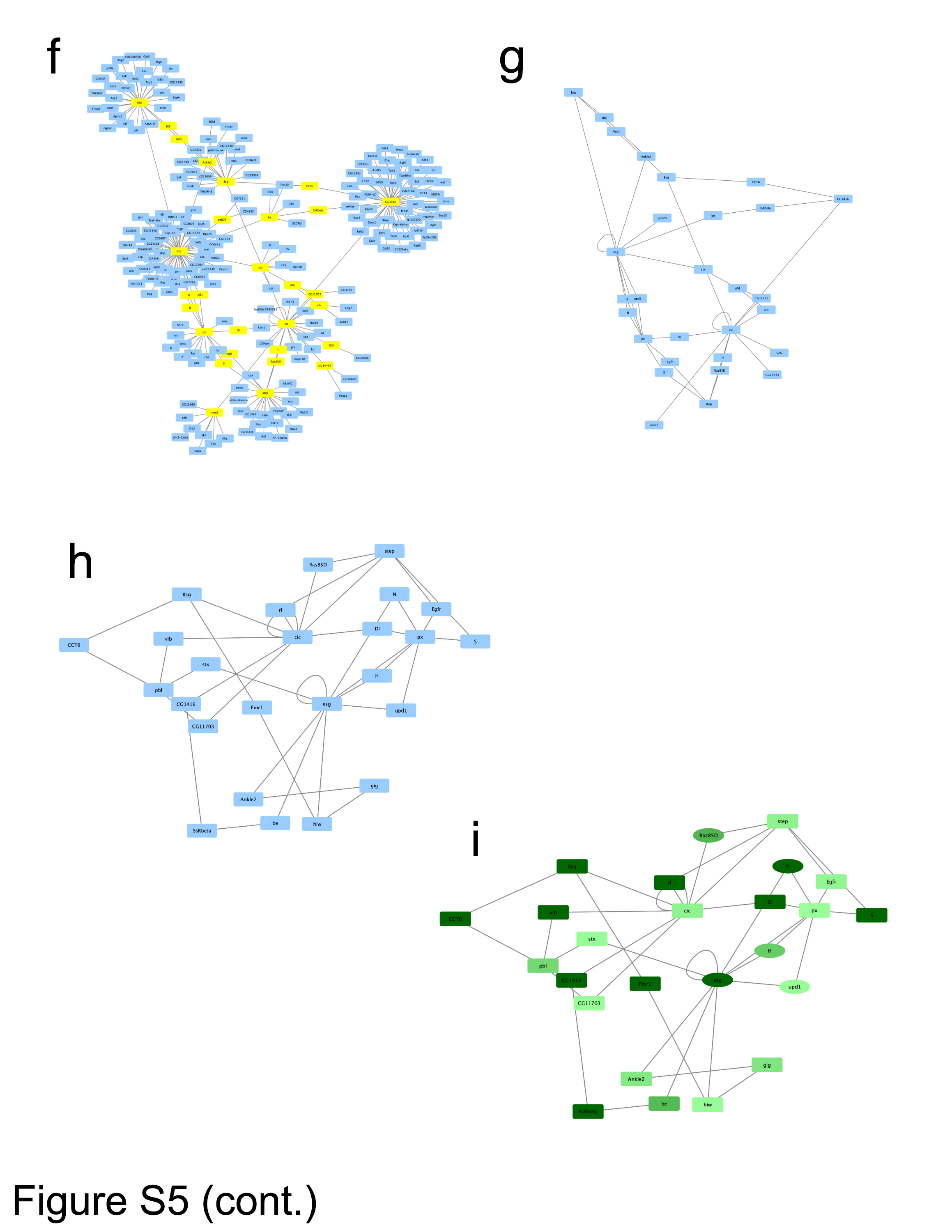

Supplement: Supplementary file 1 [file genes-10-00423-s001.zip › Supplementary Materials/Figure S5cont.jpg]

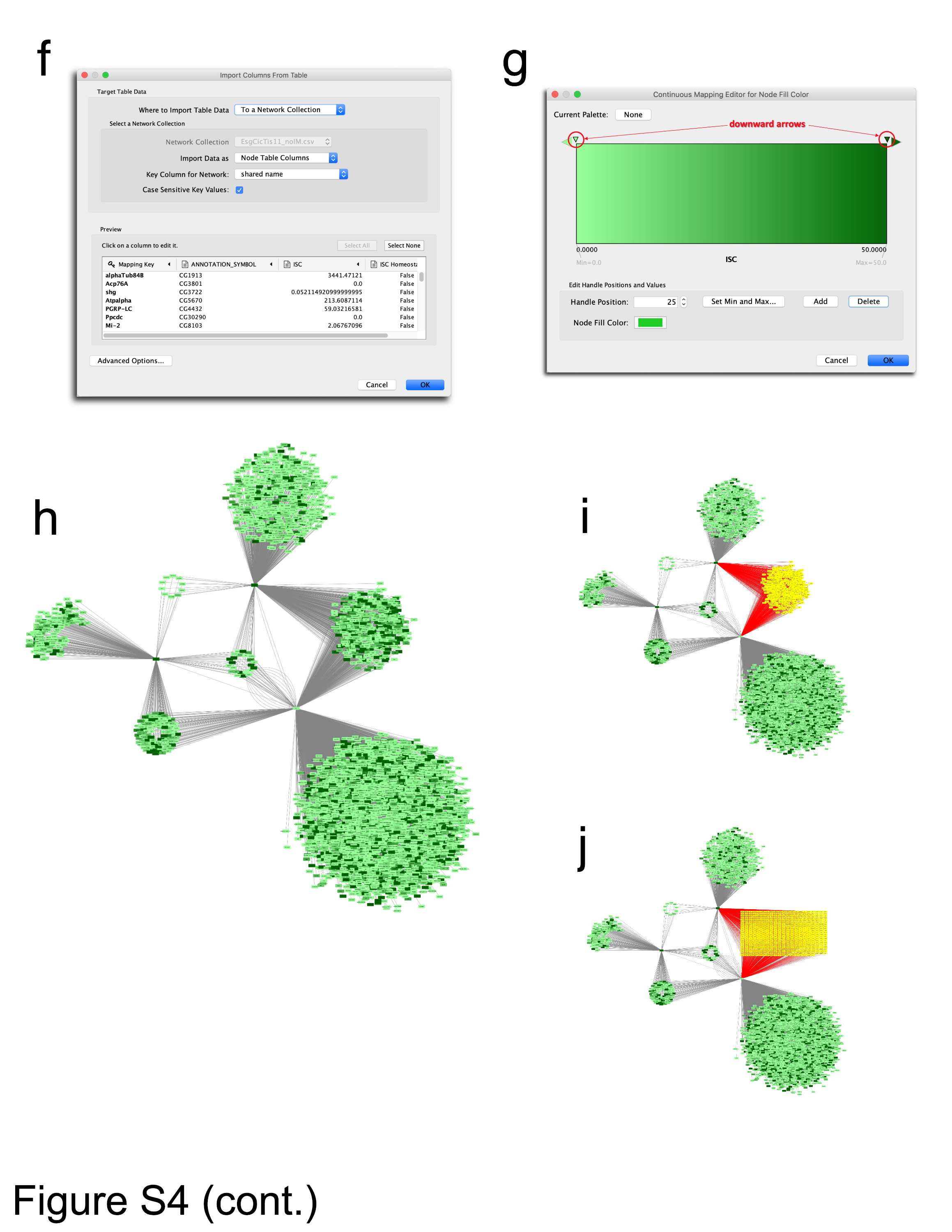

Supplement: Supplementary file 1 [file genes-10-00423-s001.zip › Supplementary Materials/Figure S4cont.jpg]

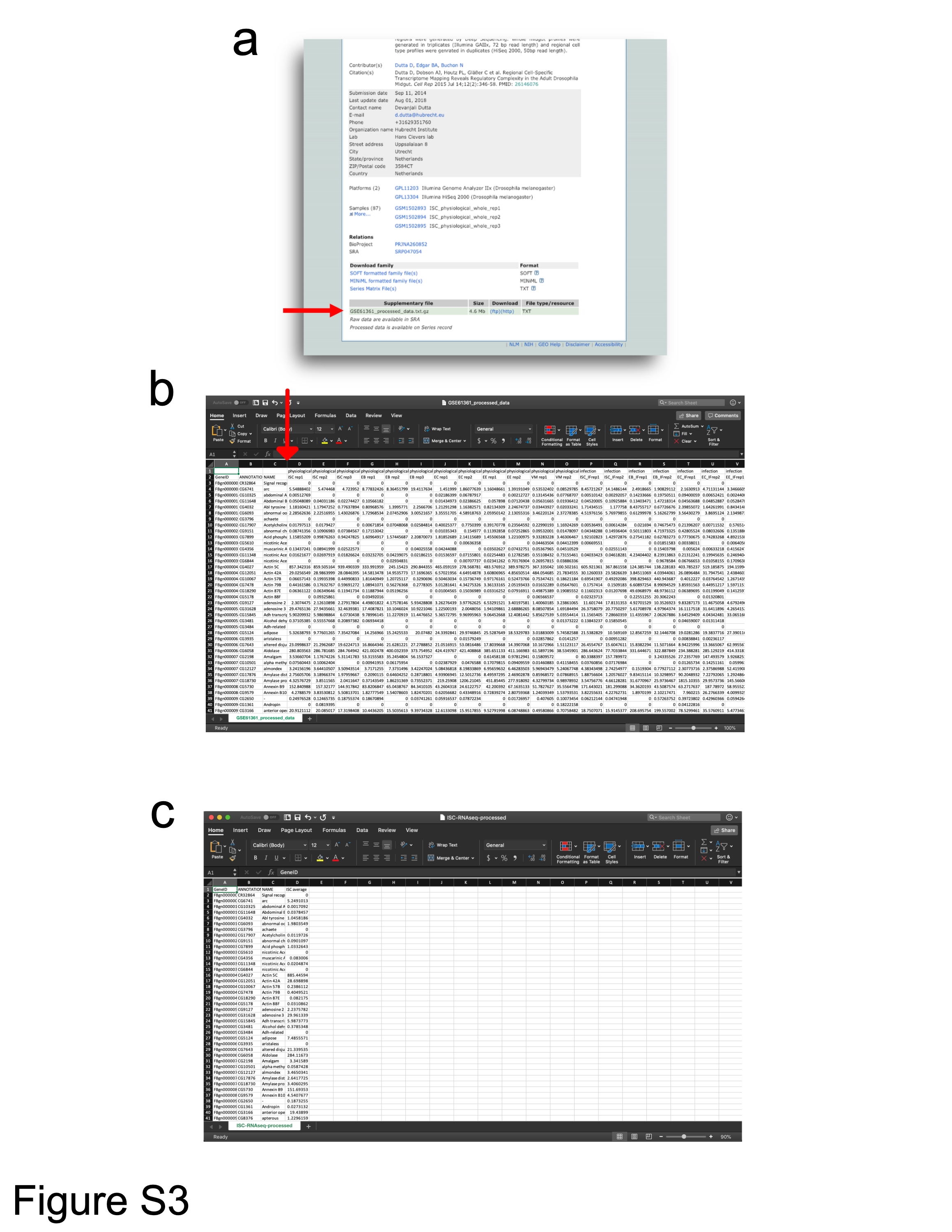

Supplement: Supplementary file 1 [file genes-10-00423-s001.zip › Supplementary Materials/Figure S3.jpg]

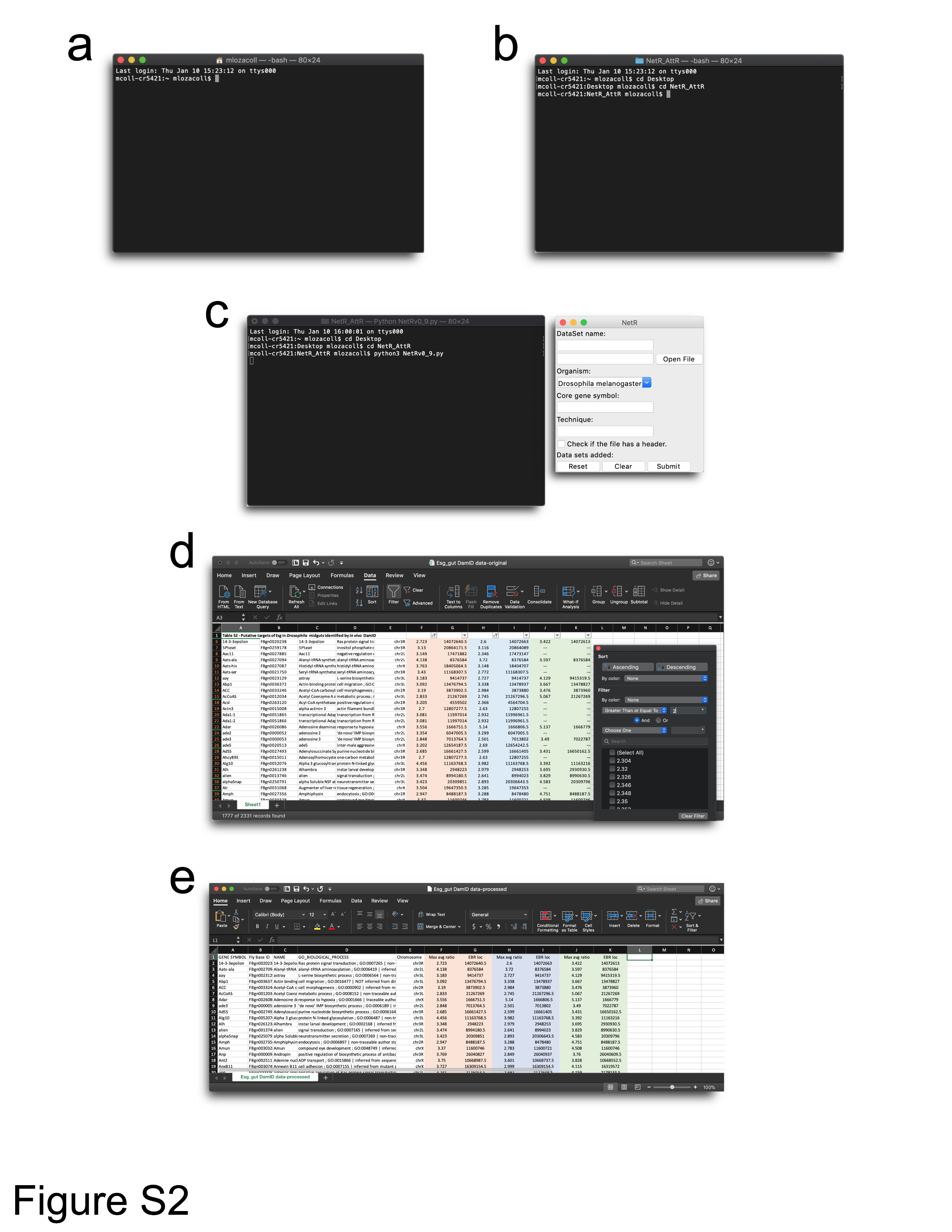

Supplement: Supplementary file 1 [file genes-10-00423-s001.zip › Supplementary Materials/Figure S2.jpg]

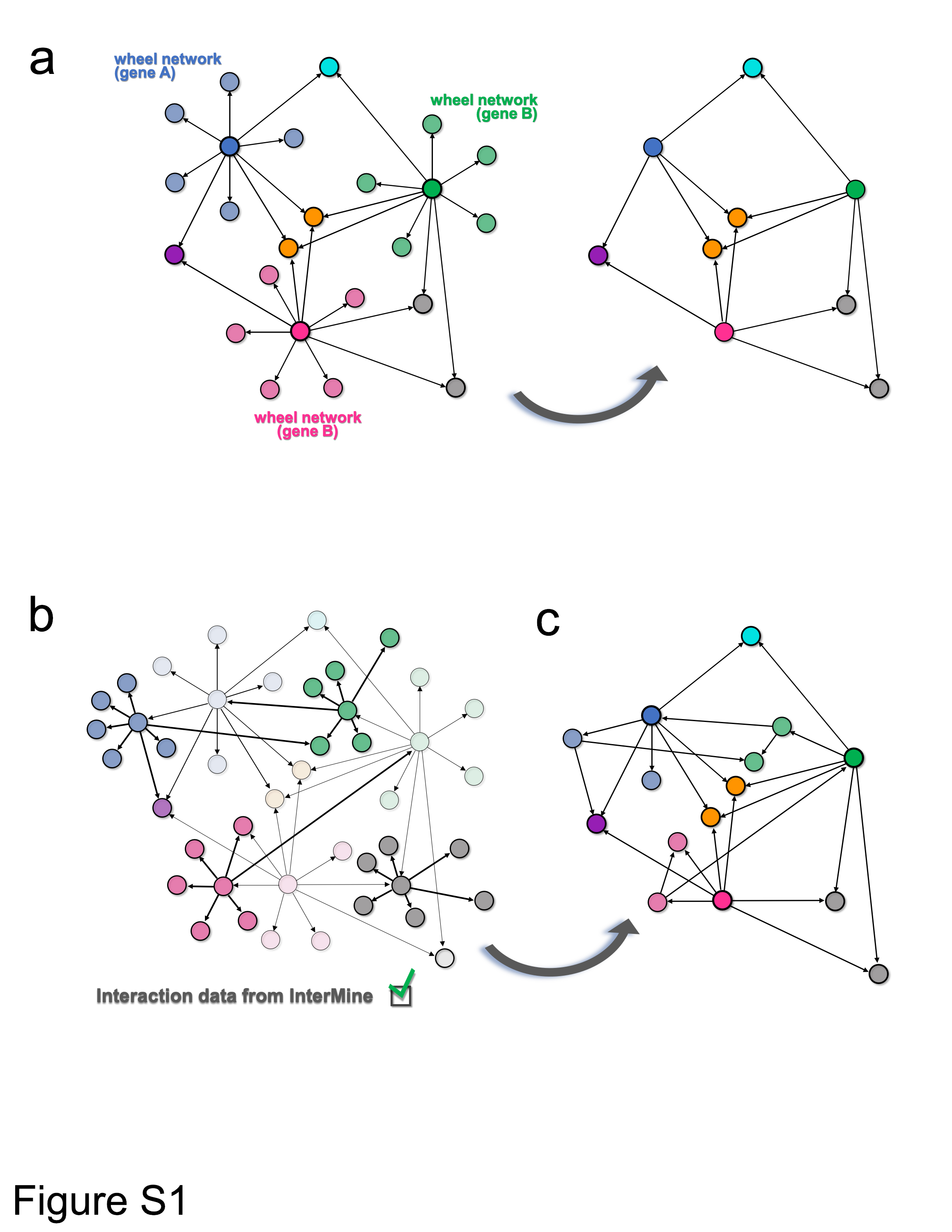

Supplement: Supplementary file 1 [file genes-10-00423-s001.zip › Supplementary Materials/Figure S1.jpg]

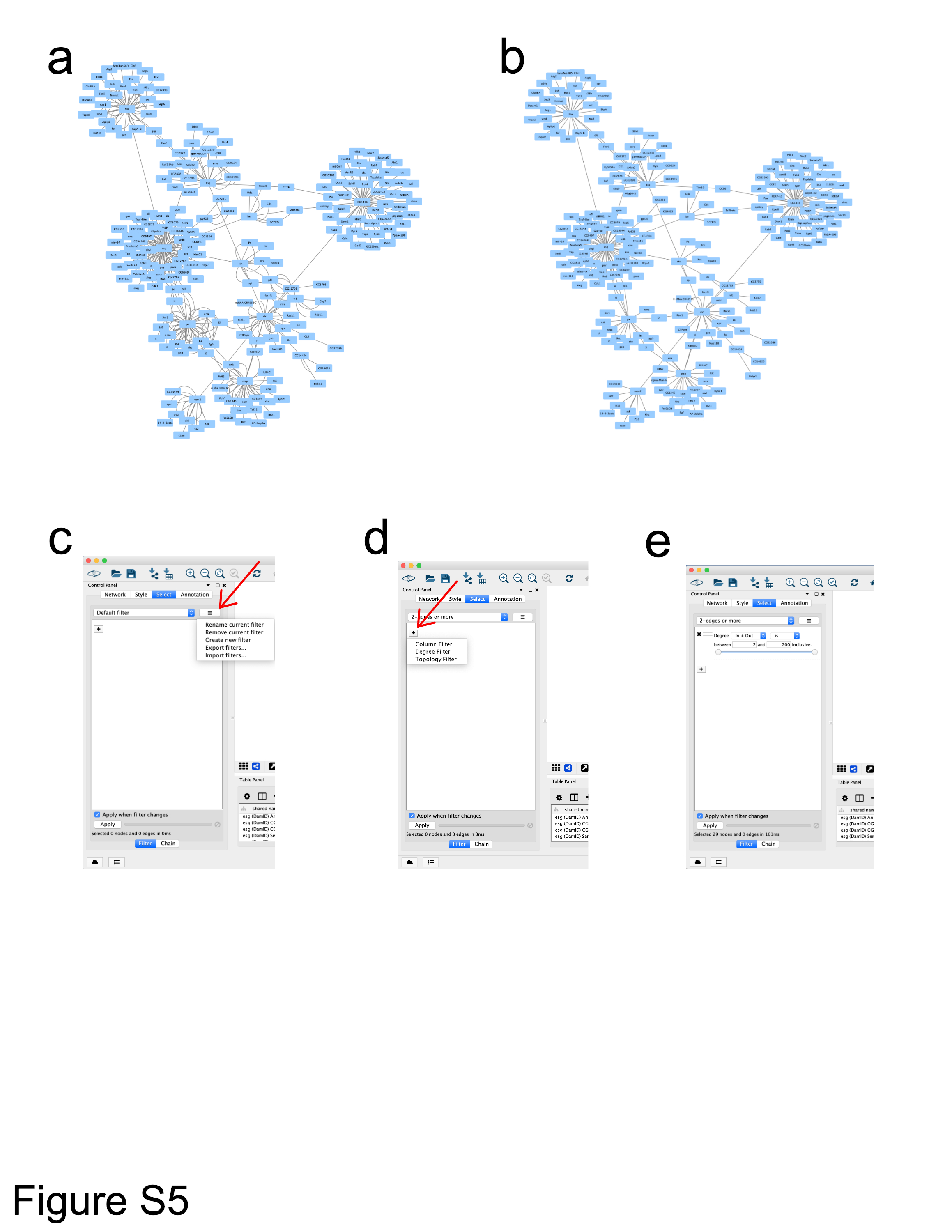

Supplement: Supplementary file 1 [file genes-10-00423-s001.zip › Supplementary Materials/Figure S5.jpg]

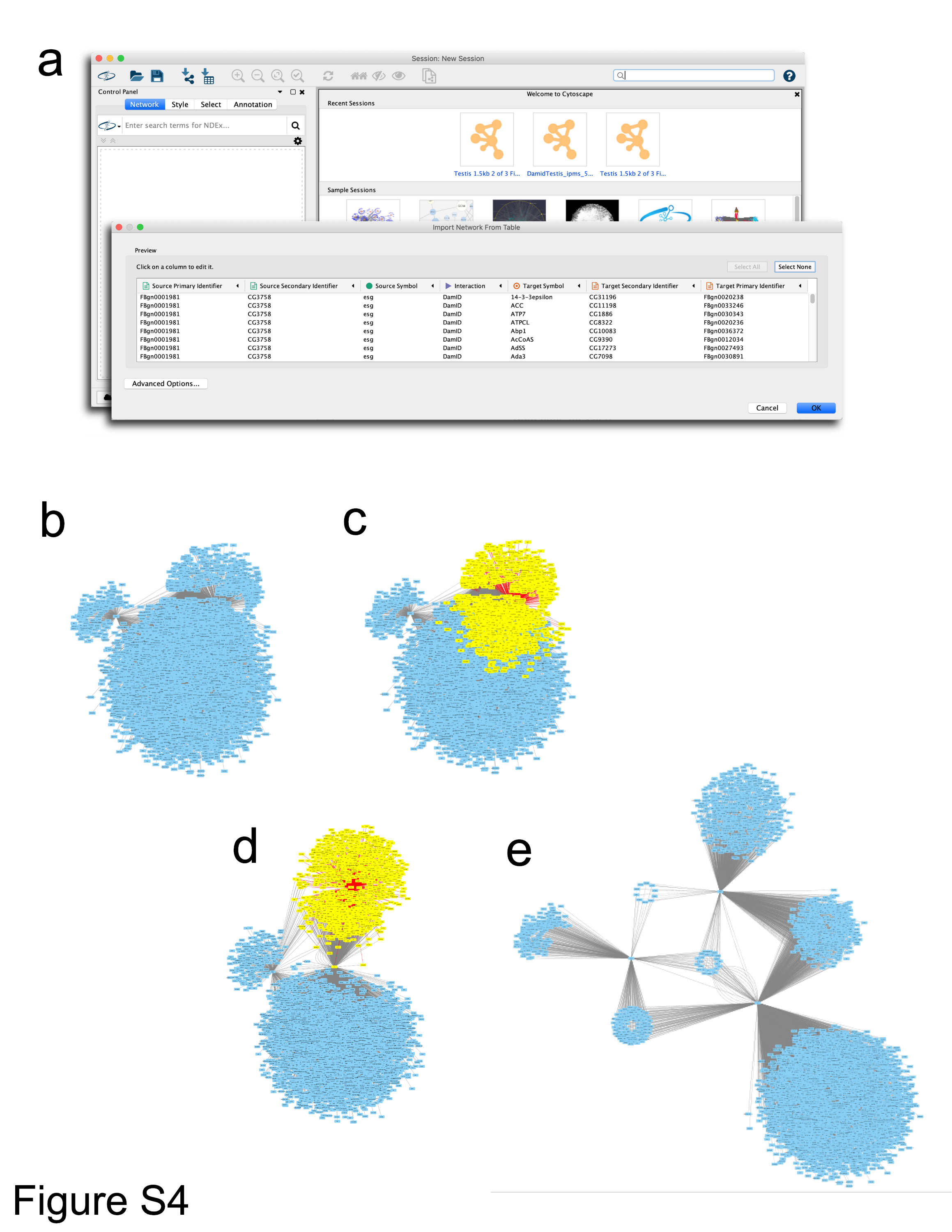

Supplement: Supplementary file 1 [file genes-10-00423-s001.zip › Supplementary Materials/Figure S4.jpg]
